# Integrated Microfluidic Devices Fabricated in Poly (Methyl Methacrylate) (PMMA) for On-site Therapeutic Drug Monitoring of Aminoglycosides in Whole Blood

**DOI:** 10.3390/bios9010019

**Published:** 2019-01-30

**Authors:** Zaidon T. Al-aqbi, Yiing C. Yap, Feng Li, Michael C. Breadmore

**Affiliations:** 1 Australian Centre for Research on Separation Science (ACROSS), School of Natural Sciences-Chemistry, University of Tasmania, Private Bag 75, Hobart, Tasmania 7001, Australia; zthal@utas.edu.au (Z.T.A.); yiing.yap@utas.edu.au (Y.C.Y.); Feng.Li@utas.edu.au (F.L.); 2 College of Agriculture, University of Misan, Al-amarah, Misan 62001, Iraq

**Keywords:** therapeutic drug monitoring (TDM), aminoglycosides, size and mobility traps (SMT)

## Abstract

On-site therapeutic drug monitoring (TDM) is important for providing a quick and accurate dosing to patients in order to improve efficacy and minimize toxicity. Aminoglycosides such as amikacin, gentamicin, and tobramycin are important antibiotics that have been commonly used to treat infections of chronic bacterial infections in the urinary tract, lung, and heart. However, these aminoglycosides can lead to vestibular and auditory dysfunction. Therefore, TDM of aminoglycosides is important due to their ototoxicity and nephrotoxicity. Here, we have developed a hot embossed poly (methyl methacrylate) (PMMA) microfluidic device featuring an electrokinetic size and mobility trap (SMT) to purify, concentrate, and separate the aminoglycoside antibiotic drugs amikacin, gentamicin, and tobramycin. These drugs were separated successfully from whole blood within 3 min, with 30-fold lower detection limits compared to a standard pinched injection. The limit of detections (LOD) were 3.75 µg/mL for gentamicin, 8.53 µg/mL for amikacin, and 6.00 µg/mL for tobramycin. These are sufficient to cover the therapeutic range for treating sepsis of 6–10 μg/mL gentamicin and tobramycin and 12–20 μg/mL of amikacin. The device is simple and could be mass produced via embossing or injection molding approaches.

## 1. Introduction

Personalized medicine requires healthcare customization with medical practices, treatments, decisions, or products specific for each patient. It is most commonly associated with diagnostic testing to detect the early onset for a change in health state. When applied to personalized therapy, it involves choosing medication based on the patient’s genetic content to identify the correct drug for treatment [1] and therapeutic drug monitoring (TDM) to ensure the correct dosing period and optimum concentration to minimize toxicity and improve efficacy [2,3]. TDM requires the monitoring of blood/plasma concentrations and is important when there is a narrow therapeutic range. TDM has been proven to progress anticancer therapies, such as methotrexate [4], as well as in new targeted anticancer agents, such as nilotinib, imatinib, sunitinib, dasatinib, lapatinib, and sorafenib [5], and is critical for immunospuressants [6]. TDM also shows benefits for a wide array of drugs, particularly antibacterials, anticonvulsants, antidepressants, antiretrovirals, antipsychotics, and β-lactam antibiotics [7]. There are a range of methods which can be used to conduct TDM, but it is typically carried out in a laboratory by using liquid chromatography mass spectrometry and immunoassays [8].

For some applications of TDM, there is a desire to either be able to test at-home, such as for patients receiving treatment for chronic conditions, or for a rapid and quick response to enable quicker and more efficient treatment. The most well-known example of TDM is measuring blood glucose levels such that a diabetic can self-dose the correct amount of insulin. There are now many different blood glucose analyzers available on the market for rapid on-site testing; however, these are predominantly based on either an antibody or an immunoassay. For targets where there is insufficient specificity in these approaches, they are usually analysed with a high resolution separation, which is much harder to miniaturise than an immunoassay. This capability has been recently demonstrated through the introduction of a portable electrophoretic device, Medimate [9], which can measure lithium levels in blood. The Medimate is an example of a point-of-care (POC) device based on the micro total analysis system (μTAS) concept [10]. A µTAS offers the possibility to integrate multiple procedures in a portable, low-cost platform which is simple to utilize without affecting the results. TDM of lithium with Medimate has been studied and recently deemed to be clinically useful [11] and is appropriate for self-testing [12]. While the Medimate device is an impressive and important development for the field, lithium is well-separated from other matrix components in the blood, and has a high therapeutic range, making detection relatively simple. Other μTAS devices applicable to TDM have been developed for glutathione [13], creatinine [14], and β-hydroxybutyrate (βHB) [15]. 

One of the main impediments in developing a µTAS is the complexity and size of the instrumentation required for the on-site analysis, and for at-home monitoring, it needs to be fully automated, simple to use, easy, and low-cost to make. Recently, Shallan et al. [16] developed a microfluidic device featuring two nanojunctions with different pore sizes to create a size and mobility trap (SMT) to purify and concentrate small molecules prior to an integrated electrophoretic separation. Significantly, the nanojunctions were created by dielectric breakdown after the device containing easier-to-fabricate micron-sized channels was bonded, providing a simpler pathway to commercial manufacture. The potential of the device for TDM was demonstrated with the determination of the antibiotic ampicillin from whole blood within 5 min. However, this device was fabricated in Polydimethylsiloxane (PDMS) where large-volume production is not possible through hot embossing or injection molding. Li et al. [17] developed a similar device incorporating two commercially manufactured nanoporous membranes for the concentration and monitoring of albumin in urine as a marker of albuminurea. While functional, the pore sizes were too large for small molecule pharmaceuticals, and the additional fabrication steps to incorporate the two different membranes would significantly increase the cost of the device. 

Here, a microfluidic device with an SMT feature created by dielectric breakdown was fabricated in PMMA instead of PDMS because of the well-known ability of hot embossing or injection molding in this material [18]. We demonstrate that similar nanojunctions can be created in PMMA using controlled dielectric breakdown and that a device with two different sized nanojunctions can be used for the analysis of aminoglycoside antibiotic drugs in whole blood.

## 2. Experimental Section

### 2.1. Materials and Chemicals

All solutions were prepared using Milli-Q water (18 MΩ, Millipore, North Ryde, Australia). PDMS (Sylgard 184) elastomer and curing agent were purchased from Dow Corning (Michigan, MI, USA). Potassium thiocyanate (KSCN) was purchased from AJAX Chemicals (Sydney, Australia). 5(6)-Carboxy-X-rhodamine (ROX), R-phycoerythrin (RPE), fluorescein, fluorescamine, bovine serum albumin (BSA), Ferric chloride (FeCl_3_), aminoglycoside antibiotics (gentamicin, amikacin and tobramycin), hexadimethrine bromide (HDMB), hydroxypropyl methylcellulose (HPMC), sodium tetraborate, di-sodium hydrogen phosphate, and sodium phosphate monobasic for buffer preparation were purchased from Sigma-Aldrich (Sydney, Australia).

### 2.2. Sample Preparation 

Fluorescamine was prepared in acetone to obtain 3 mg/mL as a stock solution. BSA was prepared in Milli-Q water at a concentration of 2 mg/mL and then labelled with fluorescamine at a ratio 3:1 in borate buffer at pH 9. ROX and fluorescein were prepared in Milli-Q water to get 25 μg/mL and 200 μg/mL solutions, respectively. KSCN and FeCl_3_ were prepared in Milli-Q water to obtain 2.5 M and 25 mM, respectively. Aminoglycosides antibiotics (gentamicin, amikacin, and tobramycin) stock solutions were prepared in Milli-Q water at a concentration of 500 μg/mL. These were then labelled with fluorescamine at a ratio of 3:1 in borate buffer at pH 9. Whole blood samples from a healthy volunteer were collected under the guidelines by the Tasmanian Health and Medical Human Research Ethics Committee, Office of Research Services, University of Tasmania (Ethics Approval Ref is H0016575) and spiked with aminoglycosides drugs (amikacin, gentamicin, and tobramycin) (stock solution) in a ratio of 10:0.1 to obtain different concentrations (2, 5, 10, 15, 20 μg/mL). The mixture was then labelled with 20 μL fluorescamine in 20 μL borate buffer pH = 9. 

### 2.3. Fabrication of Microfluidic Device

All microfluidic devices were created on a 75 mm × 50 mm × 1.5 mm piece of PMMA (RS Components, Melbourne, Australia) using the following procedures, with a schematic shown in Figure S1. First, the negative template of the device was fabricated in SU-8 using previously described processes [19,20]. Then, PDMS mixed at 5:1 (w/w) was degassed, placed on the SU-8 template, and then heated in an oven at 70 °C for at least 12 h. The positive PDMS stamp was then removed from the PMMA template and thermally aged in an oven at 250 °C for 30 min. The aged PDMS template was used to hot emboss the PMMA channel plates (1.5 mm × 50 mm × 75 mm) as follows: the blank PMMA plate and PDMS positive master were placed between two 100 mm × 100 mm × 6 mm glass plates and put into a hot embosser (MTP-8, Tetrahedron, San Diego, CA, USA). Four steps were used in the embossing process: Step 1: temperature increased to 130 °C at a rate of 92 °C /min, at a pressure of 100 lbs; Step 2: increasing the pressure up to 380 lbs at a rate of 75 lbs/min at the temperature of 130 °C, and hold for 20 min; Step 3: the temperature was reduced at a rate of 15 °C /min until 60 °C at 380 lbs; Step 4: the pressure was released, and the embossed device removed. The hot embossed PMMA device was then sealed with single-sided adhesive tape (Tesa SE, Charlotte, NC, USA), with the device’s microchannels placed downward on 1mm thick stainless steel plates. The assembly was then fed into the officer laminator (Peach 3500, Peach, Switzerland) at a 20 °C temperature, speed 5 for four passes, with 90 degree clockwise rotation at each pass. 

### 2.4. Creation of Nanojunctions by Controlled Dielectric Breakdown

**Figure 1:**
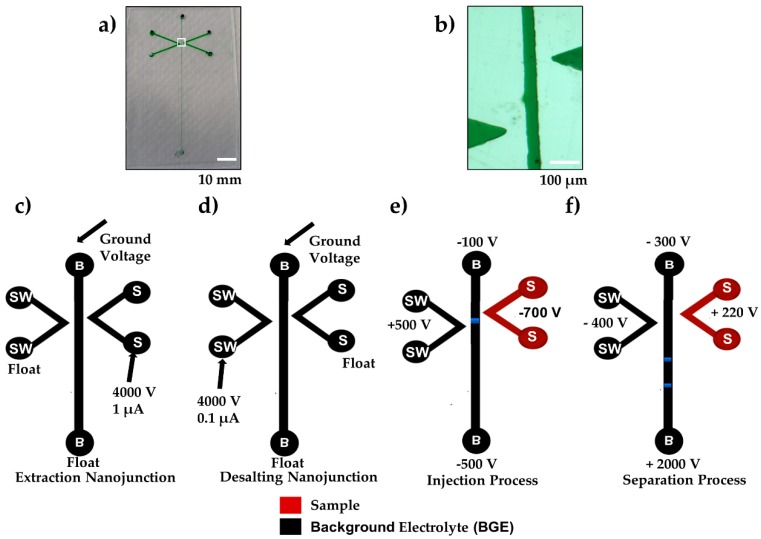
(**a**) Photograph image of the double-V hybrid PMMA/adhesive tape device filled with green food dye. Scale bar = 10 mm. (**b**) Zoomed in microscope image of the offset double-V channel filled with green food dye (white box in panel a). Scale Bar = 100 µm. (**c**) Schematic of the microfluidic device (dimension not to scale) indicating voltages and terminating current used for generation of the extraction nanojunction and (**d**) the desalting nanojunction. (**e**) Injection voltages (60 s) to extract, concentrate, and desalt injected plug (blue) of small molecules (ROX and fluorescein). (**f**) Separation voltages (180 s) for electrophoretic separation. Buffer (B), Buffer Waste (BW), Sample (S), and Sample Waste (SW).

The microfluidic device is hybrid PMMA/adhesive tape with the complete microchip shown in Figure 1a and a zoomed in image of the offset double V in Figure 1b. All microchannels had a 50 μm depth. The separation channel was 50 μm wide and the V channels were 500 μm. The hot embossed double-V PMMA device was used to create the SMT through the formation of nanojunctions in the 100 μm gap between the tips of the V-channels and the separation channel. This gap distance was selected instead of 20 μm [21] or 40 μm [22] reported in the literature, to allow for a lengthened use of higher voltages during the extraction and purification stages without inducing secondary breakdown. The 500 µm offset distance between the tips of the Vs permitted the two nanojunctions to connect and generate the SMT. The separation channel length was 60 mm and the effective length from the first nanojunction (injection point) to the detection point was 30 mm.

To make the nanojunctions, the separation channel and V-channel sample (S) were filled with the breakdown electrolyte, consisting of 10 mM phosphate buffer, pH 11, while the V-channel sample waste (SW) was filled with 100 mM phosphate buffer, pH 7. The nanochannels were created by applying a high voltage of 4000 V over 100 µm (40 V/µm, which is above the dielectric strength of PMMA 36 V/μm [23]) to the V-sample (S) channel or sample waste (SW) channel whilst the separation channel was kept grounded, as shown in Figure 1c,d. The current limit was varied from 0.1 μA to 5 μA by utilizing an in-house adjustable power supply, controlled by the LabView HV V.6 program (National Instrument, Austin, TX, USA), which terminated the voltage once the current limit was achieved. The current limits were chosen to allow the transport of different sized molecules. The V-channels and the separation channel were then refilled with the experimental solutions after cleaning using Milli-Q water. 

### 2.5. Microdevice Operation

After nanojunction creation, the sample V-channel (S) was filled with sample solution using an autopipette. The separation channel was filled with 100 mM phosphate buffer, 0.5% HPMC, or 0.1% of HDMB at pH 11.5, while the V-channel sample waste (SW) was filled with 10 mM phosphate buffer, pH 11.5, similar to those reported by Shallan et al. The extraction and separation voltages were applied using external electrodes for each reservoir, as shown in Figure 1e,f. 

Visualization of ion enrichment/depletion was conducted using a Nikon high-definition colour CCD camera head (Digital Sight DS-Fi1c, Nikon, Japan) operated with NIS-Elements BR 3.10 software (Melville, NY, USA) mounted on an inverted fluorescence microscope (Ti-U, Nikon, Tokyo, Japan). Multiband pass excitation (λ_ex_ at 390, 482, 563, and 640 nm) and emission (λ_em_ at 446, 523, 600, and 677 nm) filters (Semrock, Rochester, NY, USA) were used for all the experiments. A photomultiplier tube (PMT) (Hamamatsu Photonics KK, Hamamatsu, Japan) was used to perform the quantitative measurements which was connected to the microscope. An Agilent interface (35900E) connected to a laptop and operated by Agilent ChemStation for LC software (Agilent Technologies, Waldbronn, Germany) was used for data acquisition. An in-house four-channel (0–5 kV) dc power supply was used to apply an electrical potential to each reservoir through a custom designed interface connected to six platinum electrodes.

## 3. Results and Discussions

In our previous work, we designed and demonstrated a new electrokinetic device with a SMT for the concentration and extraction of drugs, followed by their separation by electrophoresis. Electrophoresis is a separation mode in which ions, molecules, and particles are separated in an electric field in a conducting liquid medium. The speed of separation can be manipulated by variation of the liquid media composition, but is generally dependent on the charge and size of the ion/particle. Electrophoresis is known to be simpler than liquid chromatography, the other most common separation approach for TDM, and more easily miniaturized for portability. Here, we build on our previous electrokinetic sample treatment strategy, and implement it in a more manufacturable material.

### 3.1. Nanojunction Creation and Transport Properties

**Figure 2:**
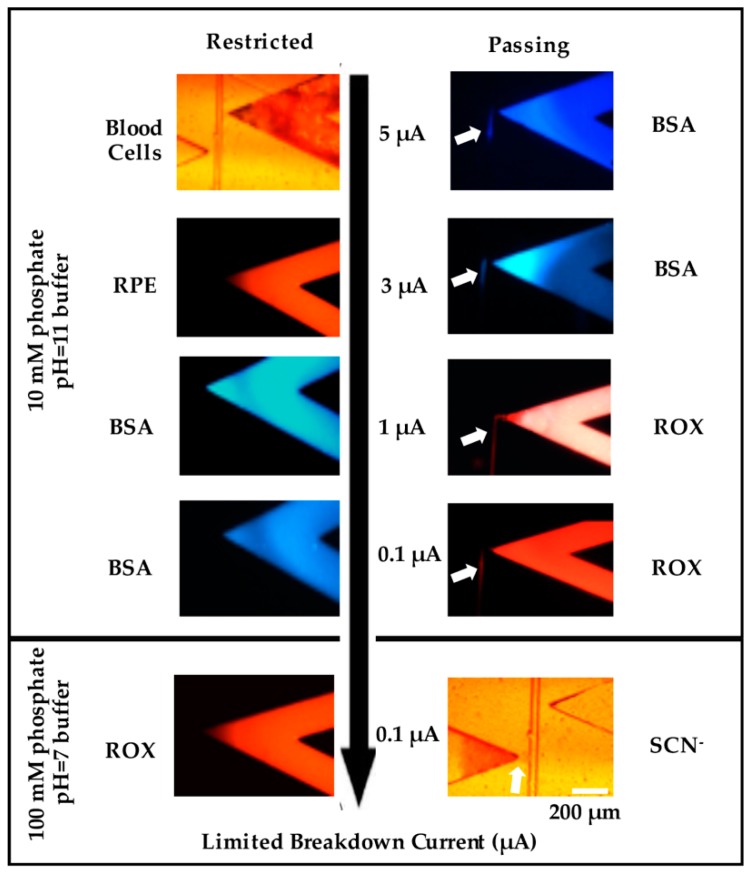
Screen shots showing the transport (right column) and restriction (left column) of different molecules through nanochannels created under different conditions. The top row shows the restriction of Blood Cells (left) and transport of BSA (blue, right); the second row shows the restriction of PDMS (red, left) and the transport of BSA (right); the third row shows the transport of ROX (right, Red) and restriction of BSA (left); the fourth row shows the restriction of BSA (left) and transport of ROX (right); the fifth row shows the restriction of ROX (left) and transport of thiocyanate (left). The first four rows used nanochannels created using a breakdown electrolyte of 10 mM phosphate buffer, pH = 11, and different terminating currents (5, 3, 1, and 0.1 μA), while the fifth row used a breakdown electrolyte of 100 mM phosphate buffer, pH = 7, and a terminating current of 0.1 μA. Images on the right show permeability (arrow), while those on the left show blocked transport. Scale Bar = 200 μm.

Previously, Shallan et al. demonstrated the ability to make nanojunctions of different sizes in PDMS by dielectric breakdown with termination currents from 1–5 μA [16,24]. Different charge and size analytes, such as labelled proteins (4–10 nm), aromatic compounds (0.5–1.0 nm), and small inorganic ions (effective hydrated radii of about 0.3 nm) [25], were used to understand whether there were differences when compared to the previous work in PDMS. The results from these experiments are summarized in Figure 2.

A single nanojunction was formed after the breakdown using different current limits. When the termination current was set at 5 μA (Figure 2 top row), the resulted nanojunctions blocked blood cells (6–8 μm) [24] and RPE (~10 nm in size) [17] from entering the separation channel, whilst electrophoretic transport of BSA labelled-fluorescamine (2–4 nm) into the separation channel was observed, with similar results obtained at 3 μA (Figure 2 second row). Reducing the termination current to 1 μA (Figure 2 third row) blocked the BSA, but allowed the transport of anionic ROX, fluorescein, and drugs. Previously, the movement of BSA was restricted in PDMS nanojunctions created with a termination current of 3 µA [24], higher than what was achieved in this work. The different behavior in the PMMA devices may be related to the difference in surface charge and/or the elasticity between the two materials. To produce smaller channels, the ionic strength of the breakdown electrolyte was increased from 10 mM phosphate to 100 mM phosphate. A termination current of 0.1 µA resulted in restricting the transport of BSA, ROX, fluorescein, and drugs, while still transporting small ions (thiocyanate) (~0.3 nm) (Figure 2 bottom row). Overall, based on the permeability results of different sized molecules (Blood cell 4–6 µm, RPE ~10 nm, BSA 2–4 nm, ROX 1 nm, inorganic ions 0.3 nm), we were able to estimate the size of fabricated nanochannels with this method. 

### 3.2. SMT Implementation

The SMT is based on the preferential electrokinetic transport of ions through the resulting nanojunctions. The extraction nanojunction was formed by applying a 1 μA termination current using 10 mM phosphate pH = 11 electrolyte buffer as this allows small ions transport (e.g., ROX) through the free transport region, while restricting cells and plasma proteins transport (e.g., BSA). The concentration nanojunction between the separation channel and the V-sample channel was formed with a termination current of 0.1 μA using 100 mM phosphate pH = 7 as this prevents the transport of target analytes (e.g., ROX), but permits the small inorganic ions (e.g., Thiocyanate). 

**Figure 3:**
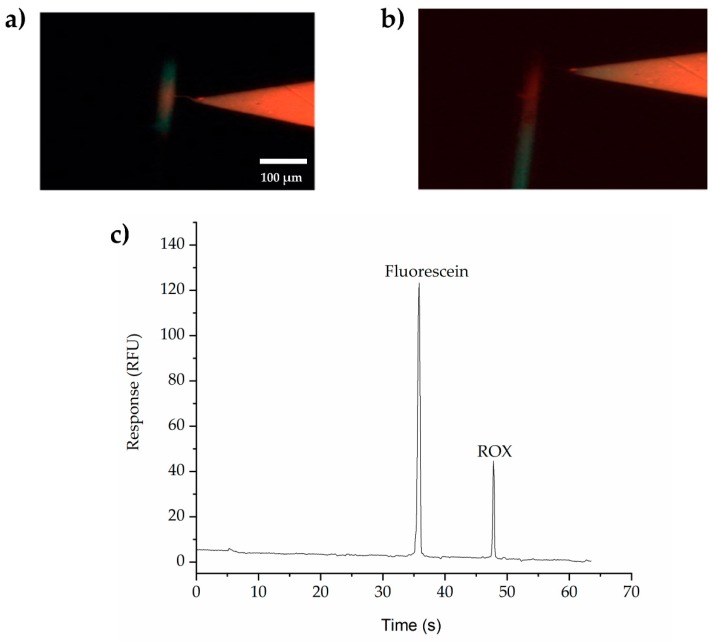
Screen shots showing (**a**) extraction, trapping, and (**b**) separation. Scale Bar = 100 μm. (**c**) Detection of small molecules (fluorescein and ROX). Current limit was set at 1μA for the right side and 0.1 μA for the left side. BGE in the separation channel is 100 mM phosphate buffer, pH 11.5, with 0.5 % HPMC to suppress the EOF and 0.1% HDMB to reverse the EOF, while in the left V-sample waste channel, 10 mM phosphate buffer, pH 11.5, was used without HPMC. Applied voltages were set at −100, −700, −500, and +500 V (for the injection) and −300, +220, +2000, and −400 V (for the separation) for reservoirs B, S, BW, and SW, respectively.

Figure 3 presents the ability and efficacy of the SMTs to extract and concentrate multiple small molecules. Figure 3a is a photo taken during the extraction and concentration of fluorescein (MW 332 Da) and ROX (MW 534.60 Da) in the separation channel. Figure 3b shows the separation of fluorescein and ROX immediately after switching the voltages from extraction and concentration to separation. 3C is the fluorescence trace collected using a PMT positioned 30 mm from the double-V intersection. These experiments show highly efficient separations of both fluorescein and ROX, with efficiencies of 414,000 and 203,000 plates/m, respectively. The relative standard deviation (RSD%) = 11.5 % for fluorescein and 4.5 % for ROX (*n* = 3 devices). 

### 3.3. Extraction and Analysis of Aminoglycosides in Whole Blood

**Figure 4:**
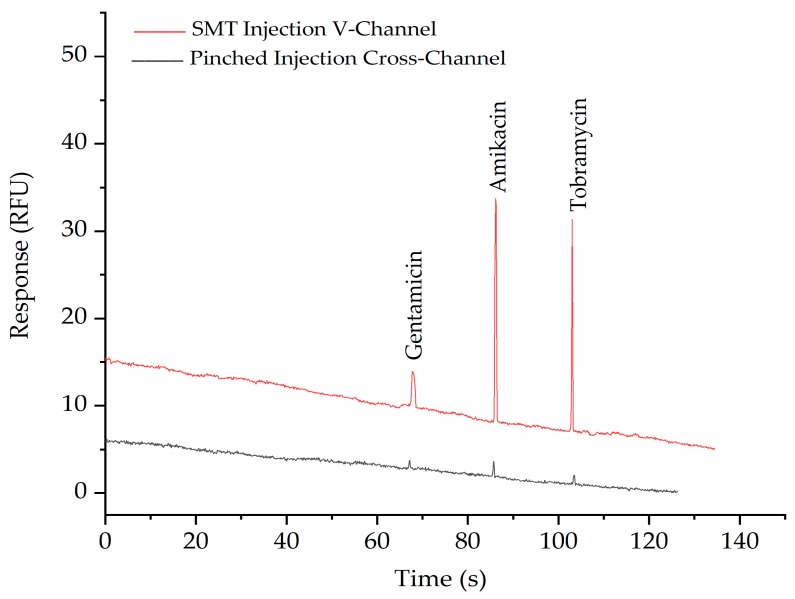
Electropherograms show the comparison of SMT (red trace) with pinched injection (black trace) of 5 ppm Gentamicin, 20 ppm Amikacin, and 10 ppm Tobramycin after labelling with fluorescamine. The BGE in the V- sample waste channel was 10 mM phosphate buffer, pH 11.5, while in the separation channel, it was 100 mM phosphate buffer, pH 11.5, with 0.5% HPMC. Applied voltages for SMT were −100, −700, −500, and +500 V for 60 s and for separation were −300, +220, +2000, and −400 V at reservoirs B, S, BW, and SW, respectively. For pinched injection (cross-channel), all channels were filled with 100 mM phosphate buffer, pH 11.5, with 0.5% HPMC. Applied voltages for injection were −100, −400, −300, and +400 V at B, S, BW, and SW reservoirs, respectively, and separation voltages were −200, +400, +1500, and +400 V at B, S, BW, and SW reservoirs, respectively.

The purpose of the SMT is to purify and concentrate anionic pharmaceuticals with a low molecular weight (<1000 Da) from biological fluids such as blood. The applicability of the PMMA-embossed device for achieving this was demonstrated by the detection of the three aminoglycoside drugs gentamicin, amikacin, and tobramycin. after derivatization with fluorescamine using both the double-V device and a normal cross channel design with pinched injection [26]. Figure 4 shows the separation of a standard mixture of these three labeled aminoglycosides in both the double-V device and a normal cross-design with pinched injection. It can be seen from the figure that the peaks in the double-V design are significantly higher than in the cross-device, demonstrating the concentration ability of the device. A 30-fold enhancement factor was achieved without any significant broadening of the peaks. 

**Figure 5:**
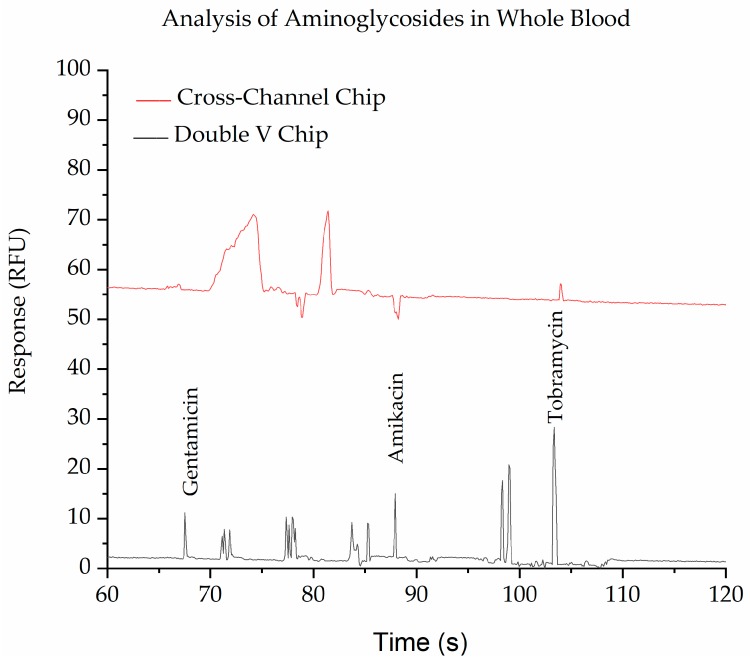
Electropherograms show the purification of whole blood spiked with 10 ppm of gentamicin, amikacin, and tobramycin and derivatized with fluorescamine using a cross-chip (red trace) and the Double V- chip (black trace). The BGE in the V-sample waste channel was 10 mM phosphate buffer, pH 11.5, while in the separation channel, it was 100 mM phosphate buffer, pH 11.5, with 0.5% HPMC. Applied voltages for injection were −100, −700, −500, and +500 V for 60 s and for separation were −300, +220, +2000, and −400 V at reservoirs B, S, BW, and SW, respectively. For cross-channel, all channels were filled with 100 mM phosphate buffer, pH 11.5, with 0.5% HPMC. Applied voltages for injection were −100, −400, −300, and +400 V at B, S, BW, and SW reservoirs, respectively, and separation voltages were −200, +400, +1500, and +400 V at B, S, BW, and SW reservoirs. respectively.

The other function of the SMT is to purify the sample. This can be seen in Figure 5 and Figure S2a, which show the separation of blood spiked with the aminoglycosides and then labelled with fluorescamine. The cross device provides a clean peak for tobramycin, but interferences are observed for gentamicin and amikacin. This is due to fluorescamine reacting with all primary amines, including those on drugs, proteins, amino acids, etc., found in the blood. In contrast, the double-V device containing the SMT provides clean separations due to the removal of some of the interferences, most likely the larger proteins. Some endogenous small molecules are still observed in the SMT, but these are well-resolved from the target drugs in this instance, allowing full and complete quantitation of the three aminoglycosides. The device shows high recovery efficiencies for the three drugs (Gentamicin 92%, Amikacin 98%, and Tobramycin 98%) and there were no clogging cases observed during the analysis. 

The linear calibration curve of the Aminoglycosides (gentamycin, amikacin, and tobramycin) from blood is shown in Figure S2b. The LOD (signal to noise level of three) was 3.75 µg/mL for gentamicin, 8.53 µg/mL for amikacin, and 6 µg/mL for tobramycin, and they are linear to 2–20 µg/mL, which covers the therapeutic range for treating sepsis of 6–10 μg/mL gentamicin and tobramycin and 12–20 μg/mL of amikacin [27].

In comparison to current methods for these aminoglycosides, they can be analyzed by immuno- assays, such as the Fluorescence Polarization Immunoassay (FPIA), Radioimmunoassay (RIA), and Enzyme-linked Immunosorbent assay (ELISA). These assays have limitations. The FPIA assay has a good reproducibility, is comparatively cheap, and is labor non-intensive [27]. Additionally, FPIA is fully automated, but demands an IMx FPIA analyzer [28] that is relatively expensive. RIA was successfully used to determine the aminoglycosides in biological samples, but it has problems related to handling radioactivity and the equipment is costly [29]. The ELISA system labeling with primary antibodies is time-consuming and the labeling of the primary antibody from one experiment to another is not flexible [30]. HPLC (High Performance Liquid Chromatography) is a sensitive method for conducting the aminoglycosides, but requires expensive instruments [31] and shortening the separation time is required [32]. By Higher Performance Liquid Chromatography (HPLC), the LOQ for tobramycin is as low 0.05 μg/mL using 200 μL of plasma [33]; however, these low levels are not necessary given the therapeutic range of aminoglycosides. When monitoring preterm infants, it is more profitable to use smaller volumes of sample, which should not overtake 75 μL [34]. Here, the suggested assay demands 20 μL of blood to give results within 3 min. 

## 4. Conclusions 

We have demonstrated an integrated hot embossed PMMA featuring an SMT device for desalting, on-chip extraction, and concentration. The hot embossed PMMA controlling dielectric breakdown enabled nanojunctions’ pore size tuning. This method permitted the analysis of aminoglycosides as negatively charged drugs which were off-chip spiked with on-site finger prick blood (~20–100 µL) and labelled with fluorescamine. The nanojunctions created using dielectric breakdown are achieved simply and quickly, and the electric field controlling pore size permits their integration without increasing the fabrication cost. The device allows a simple operation and does not rely on valves or pumps. With integrated reagents (dried fluorescamine and liquid electrolytes) and a hand-held reader, this would create a fully-portable screening device for POC analysis of therapeutics.

## Supplementary Materials

The following are available online at http://www.mdpi.com/2079-6374/9/1/19/s1. Figure S1: Schematic of the PMMA fabrication process. Figure S2: Analysis of Aminoglycosides from whole blood.
