# Supplementary material for: Integrated Microfluidic Devices Fabricated in Poly (Methyl Methacrylate) (PMMA) for On-site Therapeutic Drug Monitoring of Aminoglycosides in Whole Blood
Source: Biosensors (Basel). 2019 Jan 30;9(1):19. doi: 10.3390/bios9010019 (PMC6468438; doi:10.3390/bios9010019)
Supplement: Supplementary file 1 [file biosensors-09-00019-s001.pdf]

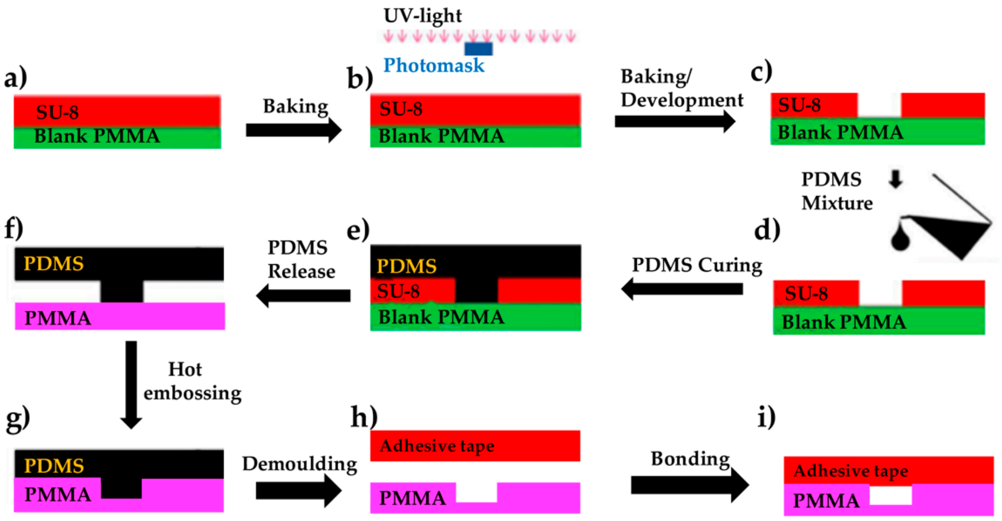

Figure S1. Schematic of the PMMA fabrication process.

(a) SU-8 2005/3025 is coated on a piece of PMMA. **(b)** The SU-8 layer is exposed to UV light and soft-baked. **(c)** The exposed resist is developed. **(d)** A mixture of (5:1) PDMS is casted onto the PMMA template. **(e)** The PDMS is placed in an oven and slowly cured. **(f)** The PDMS stamp is released and placed on a new piece of PMMA. **(g)** The PDMS and PMMA are compressed using the hot embosser. **(h)** The embossed PMMA with a microchannel is removed and a single-sided adhesive tape is used to bond it. **(i)** The final bonded PMMA-tape device.

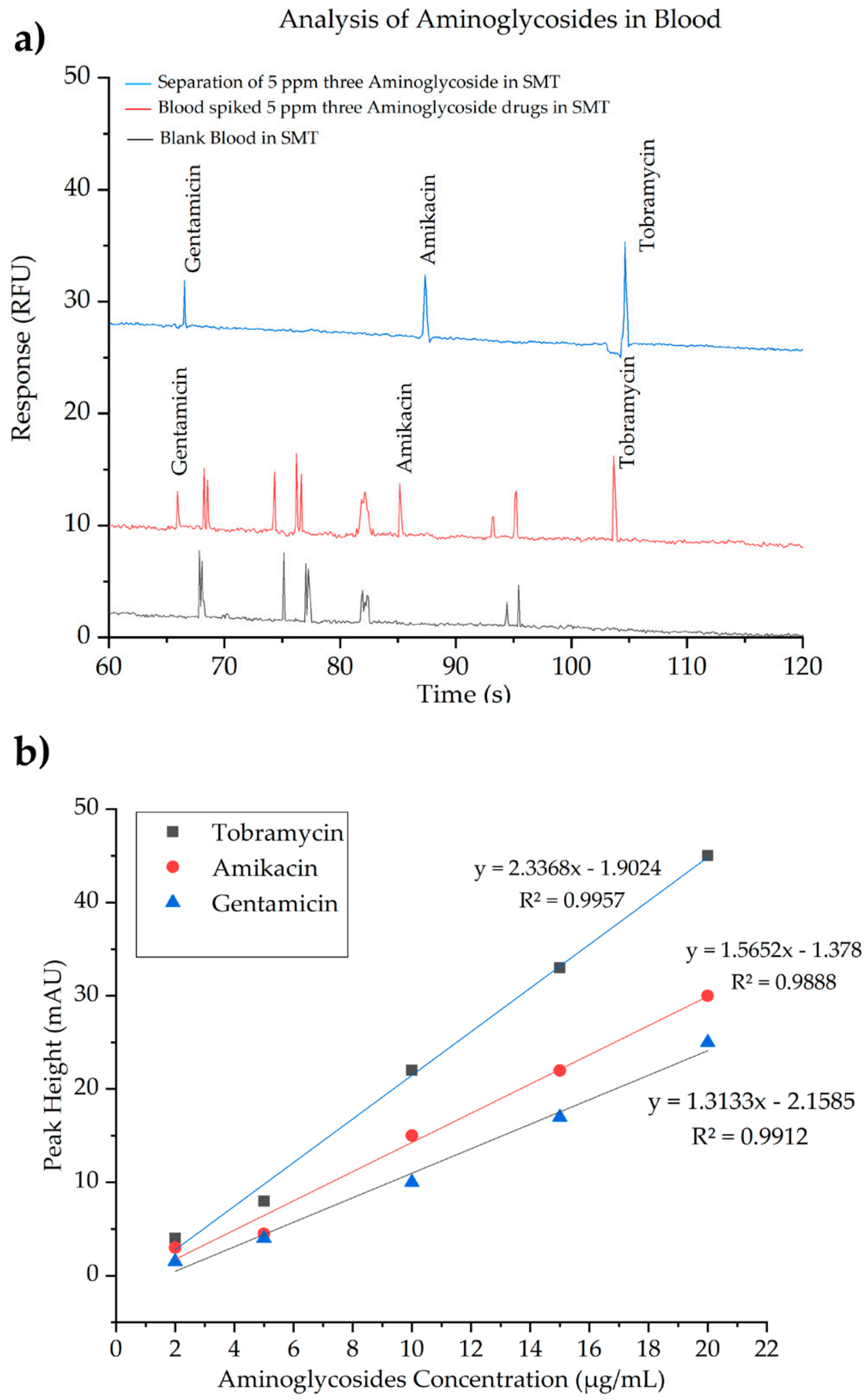

Figure S2. Analysis of Aminoglycosides from whole blood.

(a) Separation of 5 µg/mL of three Aminoglycosides in SMT (blue). Whole blood spiked with 5 µg/mL Aminoglycosides (red). Blank blood (black). **(b)** The linear calibration curve for the Aminoglycoside drugs Tobramycin (black), Amikacin (red), and Gentamicin (blue) from whole blood.
